# Morphofunctional Alterations of the Hypothalamus and Social Behavior in Autism Spectrum Disorders

**DOI:** 10.3390/brainsci10070435

**Published:** 2020-07-08

**Authors:** Andrea Caria, Luciana Ciringione, Simona de Falco

**Affiliations:** Department of Psychology and Cognitive Sciences, University of Trento, 38068 Rovereto, Italy; luciana.ciringione@unitn.it (L.C.); simona.defalco@unitn.it (S.d.F.)

**Keywords:** autism spectrum disorders, hypothalamus, amygdala, oxytocin, social cognition, social interaction, affiliative behavior, neuroimaging

## Abstract

An accumulating body of evidence indicates a tight relationship between the endocrine system and abnormal social behavior. Two evolutionarily conserved hypothalamic peptides, oxytocin and arginine-vasopressin, because of their extensively documented function in supporting and regulating affiliative and socio-emotional responses, have attracted great interest for their critical implications for autism spectrum disorders (ASD). A large number of controlled trials demonstrated that exogenous oxytocin or arginine-vasopressin administration can mitigate social behavior impairment in ASD. Furthermore, there exists long-standing evidence of severe socioemotional dysfunctions after hypothalamic lesions in animals and humans. However, despite the major role of the hypothalamus for the synthesis and release of oxytocin and vasopressin, and the evident hypothalamic implication in affiliative behavior in animals and humans, a rather small number of neuroimaging studies showed an association between this region and socioemotional responses in ASD. This review aims to provide a critical synthesis of evidences linking alterations of the hypothalamus with impaired social cognition and behavior in ASD by integrating results of both anatomical and functional studies in individuals with ASD as well as in healthy carriers of oxytocin receptor (OXTR) genetic risk variant for ASD. Current findings, although limited, indicate that morphofunctional anomalies are implicated in the pathophysiology of ASD and call for further investigations aiming to elucidate anatomical and functional properties of hypothalamic nuclei underlying atypical socioemotional behavior in ASD.

## 1. Introduction

Autism spectrum disorders (ASD) are neurodevelopmental disorders with complex and diversified pathogenesis characterized by dramatic impairment of social communication, social interaction and empathy with an estimated prevalence in the general population ranging from 1 in 100 to 1 in 54 children [1]. ASD are heterogeneous disorders with multisystem and multigenic origin, where even identical genetic variations may lead to divergent phenotypic characteristics [2]. Neuroimaging studies suggested widespread abnormalities involving distributed brain networks [3,4,5,6,7], but convincing evidences of systematic differences in brain network dynamics underlying the cognitive and behavioral symptoms of ASD are still lacking. On the other hand, an accumulating body of evidence indicates a tight relationship between the modulatory functions of the endocrine system and typical and atypical social behavior [8,9,10,11,12]. In particular, two evolutionarily conserved hypothalamic peptides, the oxytocin (OT) and arginine-vasopressin (AVP), because of their extensively documented role in supporting and regulating affiliative and socio-emotional responses [13,14,15,16,17] have attracted great interest for their critical implications in ASD. 

Animal studies revealed that OT and AVP critically mediate social and affiliative behavior [18,19,20]. In addition, administration of OT has been shown to facilitate protective and nursing behavior toward pups [21]. In non-human mammals, OT is generally observed to facilitate approach behavior by decreasing avoidance of proximity and reducing defensive behavior, whereas AVP appears to modulate aggressive responses in relation to pair bonding and mating behavior, especially in males [22,23]. In humans, the effects of intranasal OT administration indicate a reduction of social stress and anxiety facilitating positive social approach and interaction, and affiliative behavior [24,25,26,27]. Moreover, intranasal AVP administration in humans, similarly to the effects observed in animals, has been shown to differentially influence social behavior in males and females, with increasing aggressive and agonistic responses in men and facilitation of pair bonding in women [28]. Several investigations also reported an association of the levels of peripheral OT and oxytocin receptor (OXTR) polymorphisms with the diagnosis and severity of ASD [29]. Genomic and epigenetic evidences for OXTR deficiency have been also observed in individuals with ASD [30]. Remarkably, a large number of controlled trials indicated that intranasal OT and AVP administration can ameliorate social abilities in autism [31,32,33,34,35,36].

Altered OT and AVP synthesis and release appear to be among the core dysfunctions underpinning the impairments in social and communication behavior of individuals with ASD [9,11,37], although it remains unclear whether OT neuropeptide can be used as biochemical marker for ASD [38].

OT and AVP are synthesized by magnocellular neurons of the supraoptic and paraventricular nuclei of the hypothalamus that secret them into the peripheral blood circulation through the posterior pituitary gland. Importantly, these peptides also act as neurotransmitters through the dendritic terminals of magnocellular neurons that release them into the hypothalamic extracellular fluid [39], and through parvocellular neurons projections to brainstem and subcortical regions, such as the amygdala, nucleus accumbens and hippocampus [40,41]. In addition, besides passive diffusion in brain circuits following dendritic release [42,43], OT transmission is also mediated by widespread long-range axonal projections of hypothalamic OT neurons [14] permitting direct modulation of the amygdala and other forebrain regions [20]. Correspondingly, OT and AVP receptors have been localized in various brain regions including the hypothalamus and the limbic system [30,44,45]. Notably, differential OT release mechanisms through dendritic and axonal terminals characterize hypothalamic activity. In fact, dendritic OT release can occur with no spiking activity, and thus, no secretion into the peripheral circulation; vice versa, electrical activity of the cell bodies can induce OT release from axon terminals without central OT release from the dendrites [46,47]. Moreover, dendritic release can lead to a very large disproportion between the concentration of OT in the extracellular fluid of the hypothalamic supraoptic nucleus and that in the periphery by over 100-fold greater [41].

Furthermore, there exists long-standing evidence of severe socioemotional impairment after hypothalamic lesions, involving in particular the ventromedial nuclei [48]. Rage has been observed after ventromedial hypothalamic lesions in both animals and humans (Wheatley 1944 [49]; Reeves & Plum 1969 [50]). Separation-induced distress vocalization can be elicited by electrical stimulation of the medial hypothalamus in guinea pigs (Herman & Panksepp 1981 [51]). Stereotactic stimulation studies in humans showed altered sexual behavior triggered by accidental focal lesions of rostromedial basal forebrain structures including the septo-hypothalamic area [52]. In addition, hypothalamic stimulation can also induce pleasurable experiences and prosocial behavior in humans [53,54]. For instance, several investigations demonstrated reduced aggressive behavior and increased social interactions after deep brain stimulation of the posteromedial hypothalamus [55]. 

Nonetheless, despite the unquestionable key role of the hypothalamus in the production of the OT and AVP (Swanson and Sawchenko, 1983), the severe socioemotional dysfunctions caused by hypothalamic lesions, and the apparent association between hypothalamic neuropeptides and socio-affective responses in ASD and neurotypical population (NT), hypothalamic involvement remains elusive in most of neuroimaging investigations exploring the neural correlates of normal and abnormal human socioemotional behavior [56,57,58,59,60,61,62]. In particular, a surprisingly limited number of studies analyzed the implication of the hypothalamus in the social impairment of individuals with ASD. 

Building on the above mentioned evidences, this review aims at providing a synthesis of neuroimaging investigations reporting morphofunctional alterations of the hypothalamus in ASD through the examination of data from individuals with ASD as well as from healthy carriers of genetic risk variation in OT receptors, as several polymorphisms of OT receptor genes have been associated with modulation of socioemotional responses and ASD [63,64,65,66,67,68,69,70,71]. A description of MR-based anatomical studies reporting abnormal morphology of the hypothalamic region will be followed by a survey of the few existing task-based and resting state functional MRI studies reporting hypothalamic alterations in individuals with ASD and in healthy carriers of genetic risk variation in OT receptors. A critical discussion integrating anatomical and functional findings will then attempt to provide some interpretations of the possible role of the hypothalamus, and its functional exchanges with cortical and subcortical networks, in the atypical socioemotional responses of ASD individuals. In conclusion, some fundamental open questions aiming at elucidating the morphological and functional hypothalamic anomalies and their impact on social cognition and behavior in ASD will be proposed.

## 2. Literature Search

This review is based on a Pubmed and Scopus search aiming to comparatively analyze the current literature until April 2020 using the following keywords “autism” AND “hypothalamus” AND “social.” In total, 236 papers were obtained from Scopus, whereas only 22 papers from Pubmed. After refining the search by limiting articles that included the term “MRI,” 42 documents remained in Scopus and just one in Pubmed. The remaining publications were then further screened for articles reporting original research studies. Careful inspection of papers, aiming to identify anatomical and functional investigations related to ASD, led to additional rejections of few unrelated papers as well as inclusion of some others missing in the initial literature search, and surprisingly resulted in only 10 relevant scientific publications for our qualitative analysis. 

### 2.1. Structural MRI Studies

One of the first direct evidence linking anatomical alterations of the hypothalamus with ASD was provided by a study assessing structural MRI based measures of brain morphometry in children and adolescents with ASD (*n* = 52) [72]. ASD individuals with respect to typically developing controls showed significant decrease of gray matter (GM) volume in the hypothalamic region including the supraoptic and paraventricular nuclei, independently of age, IQ or gender. No differences were observed in global volumes of GM, white matter and cerebrospinal fluid. 

In another study, hypothalamic atrophy was measured in young male adults with ASD (*n* = 10) with respect to neurotypical participants using two complementary structural analysis approaches [73]. First, an ROI-based voxel-based morphometry (VBM) analysis applied to the hypothalamic region, delineated through manual segmentation and including voxels in the third ventricular space between the left and right hypothalamus, revealed reduced GM density of the hypothalamus and increased cerebrospinal fluid density in the third ventricle proximal to paraventricular nucleus. Second, an automatic method was applied to a larger cohort of male ASD individuals (*n* = 41) to estimate ventricular volume of the third ventricle. This method aimed to indirectly validate previous results on the basis of the assumption that relative increase of third ventricle would imply volume reduction of the adjoining tissues. This analysis demonstrated an increase of third ventricle volume that was independent of the lateral ventricles (used as covariate), and thus excluded global brain volume increase.

Recently, decreased volume in the bilateral hypothalamus along with increased volume in the left amygdala and left hippocampus was observed in young children with ASD (*n* = 14, mean age = 4.5) compared to typically developing children (*n* = 14, mean age = 4.1) [74]. In addition, the authors observed that the hypothalamic volume was positively correlated with plasma AVP concentration. 

In parallel, several indirect evidences of abnormal hypothalamic structure and function in ASD emerged from studies of healthy OXTR risk allele carriers, in particular with the OXTR variant rs53576 that appears to be associated with lowered socioemotional responses [63,75] and is often observed in individuals with ASD [76,77,78,79,80]. 

One of the first demonstrations in this direction was a multimodal neuroimaging genetics approach that permitted to identify several neural alterations in a large sample (*n* = 212) of healthy Caucasian OXTR risk allele carriers [64]. Tost et al., using VBM, revealed a significant decrease of hypothalamic GM volume in rs53576 risk allele carriers that correlated with the degree of allele risk. Notably, decreased hypothalamic volume was predictive of a lower prosocial temperament trait in males. Structural correlation analysis, information that has been shown to mirror anatomical connectivity, showed allele-dependent increase of coupling between the hypothalamus and higher-order limbic processing areas, such as the dorsal anterior cingulate cortex, including the paracingulate cortex and amygdala (encompassing high density OT receptors), in rs53576A allele carriers.

In a consecutive study using VBM methods, reduction of GM volume in the dorsal anterior cingulate gyrus and hypothalamus was also associated in carriers of OXTR rs2254298A, another identified genetic risk variant for ASD; this result was mainly related to male carriers [81]. Structural covariance analysis revealed a significant increase in the structural connectivity between hypothalamus and dACG in rs2254298A carriers, similar to that observed in rs53576A carriers. The observed increase of anatomical coupling in healthy carriers of genetic risk variants for ASD may suggest abnormal connectivity related to alterations of several white matter morphological properties as well as atypical functional interactions [82,83].

Additional studies examining brain morphology in individuals with single nucleotide polymorphisms in the OXTR gene related to ASD indicated other alterations of locale brain volumes including the hypothalamus. Inoue et al. [84], adopting a manual tracing methodology for measuring regional brain volume, observed larger bilateral amygdala volume in Japanese adult carriers of OXTR rs2254298A, proportional to the dose of this allele. No significant association of this genotype was instead observed with hypothalamus as well as with global brain volume. In a subsequent analysis on the same data using VBM, stimulated by result of Tost et al. (2011), the same authors reported that rs2254298A was also associated with reduced GM volume in the dACG but not in the hypothalamus and amygdala [85]. However, they observed an interaction effect between gender and rs2254298A genotype in the right hypothalamus, reflecting smaller right hypothalamus volume in females only. 

### 2.2. Functional MRI Studies

Aoki et al. in a focused metanalysis of 13 fMRI studies in ASD individuals during emotional-face processing (considering both emotional-face vs non-emotional-face and emotional-face vs non-face contrasts) observed abnormal functioning of several subcortical regions [86] among which hypothalamic hypoactivity was prominent. In particular, individuals with ASD (*n* = 226, age ranging from 9 to 37 years) in comparison to NT controls (*n* = 251, age ranging from 9.2 to 28.6) showed significant hypoactivation of the hypothalamus, and hyperactivation of the bilateral thalamus, bilateral caudate, left cingulate and right precuneus. The comparison of emotional-face to non-face conditions showed a similar activation pattern but hypoactivity was also observed in the parahippocampal gyrus and amygdala, in addition to the hypothalamus. In line with behavioral studies demonstrating impaired emotional-face processing in ASD [56], the observed alteration of subcortical rather than cortical regions during face perception suggested dysfunctional unconscious processes in relation to social cognition. Notably, reduced hypothalamic activity was not observed in each of single studies included in the metanalysis, possibly because of their limited statistical power [87]. 

Preliminary evidence of a direct association between hypothalamic dysfunction and social interaction was also shown by Chaminade et al. that measured fMRI-based brain responses in ASD individuals (*n* = 10, mean age 21) during a more realistic and entertaining social behavior consisting of an interactive videogame of the popular “stone-paper-scissors” game [88]. ASD and NT participants played against three different agents: a human being, a humanoid robot endowed with artificial intelligence attempting to win the games by considering previous games’ results, and a computer that randomly generated the three possible responses. A significant interaction effect between Agent (Human, Robot) and Group (ASD, NT) delineated an activation cluster in the left and right hypothalamus, attributed to the paraventricular nucleus, resulting from decreased activity when ASD participants played against the human as compared to the artificial agent, with respect to NT. In addition, functional connectivity analysis of the left hypothalamus revealed a single cluster in the left temporoparietal junction resulting from the interaction effect of Group and Agent. Specifically, a significant negative coupling between the left hypothalamus and left temporoparietal junction (lTPJ) was measured when NT played against the robot and when ASD participants played against the human. Moreover, the coupling observed when ASD participants played against the human, but not against the robot and computer, was negatively correlated with the severity of autistic symptoms measured with Autistic Spectrum Quotient [89]. Interestingly, the decreased modulation of hypothalamic nuclei activity along with negative functional connectivity between hypothalamus and lTPJ, a region associated with anthropomorphization—which is the tendency to attribute human traits to artificial agents—was observed when ASD individuals interacted with a human player, and similarly when NT individuals played against the robot. The anticorrelation between lTPJ and hypothalamus might reflect inhibitory activity exerted by the lTPJ on hypothalamic nuclei that would then result in reduced social motivation and reward for human interactions in ASD.

In line with hypothalamic functional alterations in ASD during processing of emotional expressions [86], reduced hypothalamic activation was also observed in adult carriers of risk OXTR gene mutation for autism [64,81]. Tost et al. (2010), besides abnormal anatomy of the hypothalamus, reported functional alteration of hypothalamic activity during perception of facial expressions (using a Face-Matching Task). In particular, they observed increased fMRI-based connectivity (measured with cross correlation) between hypothalamus and amygdala, and decreased amygdala activation in adult carriers of rs53576A (*n* = 228) with respect to individuals with the GG genotype [64]. In a subsequent analysis the same authors observed reduced deactivation of the dorsal anterior cingulate and paracingulate cortex associated with healthy carriers of another OXTR gene polymorphism, the rs2254298A [81]. Moreover, differential functional brain connectivity was revealed by genotype-by-sex interaction effect associated with negative coupling of the hypothalamus with dACG and amygdala in male rs2254298A carriers, and positive coupling in females.

Likewise, Wang et al. (2013) demonstrated gender dependent effects of OXTR rs53576 gene variation on hypothalamic functional connectivity in healthy individuals. Specifically, whole brain analyses of local functional connectivity density (FCD) during resting-state fMRI data (*n* = 270) revealed a main effect of genotype on the local FCD in the hypothalamus and no gender-by-genotype interaction effect, although local FCD in male AA homozygotes was significantly lower than in male G-allele carriers [90]. Additional analysis of gender-by-genotype interaction considering resting-state functional connectivity of the hypothalamic region only showed significantly weaker coupling between the hypothalamic region and the left dorsolateral prefrontal cortex in male AA homozygotes with respect to male G-allele carriers. 

## 3. Discussion

Building on the well-recognized role of the hypothalamus in the production of the OT and AVP, and the emerging evidences of an association between activity of hypothalamic neuropeptides and socioaffective responses in ASD and NT population, we here aimed to inspect the current neuroimaging literature in humans in search for evidences of hypothalamic alterations in relation to the core social deficits in ASD. Examination of current structural and functional MRI studies reporting alterations of the hypothalamus in ASD, although rather limited, revealed quite consistent morphofunctional abnormalities. Specifically, two main findings emerged from VBM and fMRI analyses, in both adults and children: anatomical hypothalamic atrophy and functional hypoactivation during face processing and social interaction, respectively. 

### 3.1. Hypothalamic Morphological Alterations

Anatomical hypothalamic atrophy was mainly related to smaller hypothalamic volume in both ASD individuals [72] and healthy carriers of OXTR genetic risk variant for ASD, and to reduced GM density observed in ASD [73]. Notably, in line with gender-dependent differences in the expression of the OXTR gene [91,92], hypothalamic structural abnormalities in healthy carriers of OXTR genetic risk variant for ASD appear not equivalent in males and females and dependent on OXTR variants [64,85]. 

Sexual dimorphisms of the hypothalamus might follow similar gender-related differences observed in other brain regions including the amygdala, as well as in interhemispheric connectivity, along with differences in hormone-related personal traits, cognition, behavior and psychiatric disorders manifestation [93], ultimately mirroring ASD prevalence that appears larger in males than in females with a male-to-female ratio closer to 3:1 [94]. 

The observed anatomical abnormality of the hypothalamus is in line with several neuroimaging observations that, although not always congruently, reported morphological changes in ASD in multiple brain regions [95,96], including reduced volume in the social brain network [97,98,99,100]. 

However, it remains difficult to infer the exact neuronal mechanisms leading to hypothalamic atrophy, since variations of multiple properties of GM can equally affect VBM signal. Changes at the level of neuronal cell bodies, glia or neuropils might all contribute to hypothalamic grey matter reduction and differentially affect regulation of central neuropeptides and peripheral hormonal regulation through abnormal synthesis and release. Indeed, postmortem brain analysis in ASD highlighted various anatomical anomalies related to neuronal density and size, dendritic spine density, glia and cerebral vasculature [101]. In particular, lower neuronal density has been measured in human brain regions involved in social behavior such as the fusiform gyrus and amygdala [102,103,104], as well as in specific layers of ACC [105], possibly reflecting specific hypoactivation of these same regions in ASD. 

Moreover, some insights about neural mechanisms underlying hypothalamic atrophy might arise from animal models of ASD. Genetically modified animal models such as the Black and Tan Brachyury (BTBR) mouse model [106,107] and the copy number variants mouse model simulating the 15q11-13 duplication in human (15q dup) [108] were also associated with decreased GM volume of the hypothalamus. In addition, mice carriers of neurexin gene mutations have been associated with fewer oxytocin-expressing neurons in the hypothalamic paraventricular nucleus [109]. Similarly, mice with missense heterozygous mutation in the contactin-associated protein-like 2 (CNTNAP2) were characterized by specific reduction in the number of OT expressing cells in the paraventricular nucleus in association with reduced OT concentrations in brain extracts [110]. In humans, reduced plasma concentration of OT has been indeed measured in ASD [111] and predicted social impairment [29], but no clear evidences of alterations at central level emerged. Some indications suggest a possible correlation between plasma and CNS OT concentrations, but this correspondence seems particularly dependent upon the assessing methods employed [112]. Thus, there are currently no demonstrations of the specific impact of hypothalamic atrophy on OT transmission to brain circuits in humans. 

### 3.2. Hypothalamic Functional Alterations 

FMRI studies in ASD revealed hypoactivation of the hypothalamus in relation to face processing, and during interactive play with humans. Likewise, reduced hypothalamic activity during face perception was also observed in healthy carriers of risk genetic mutations for ASD [64]. 

As for morphometric anomalies, no direct interpretation of the neuronal processes underlying hypothalamic fMRI hypoactivation is yet possible. Decreased BOLD response does not necessarily imply reduced OT/AVP release. Although dendritic and axonal neuropeptides release is generally enhanced by increased action potential frequency, the BOLD signal neither directly nor exclusively reflects neuronal spiking activity but correlates more strongly with local field potentials, which represent postsynaptic activity and integrative soma-dendritic processes [113]. Considering the observed possible uncoupling between hypothalamic spiking activity and dendritic oxytocin release, which can be locally mediated by intracellular calcium stores independently of action potentials [114], decreased BOLD signal in the hypothalamus might indeed reflect reduced dendritic release of oxytocin. 

Animal models of ASD indicate some convergent evidences in relation to the hypothalamic activity. For instance, 15q dup mice showed no hypothalamic activation in response to odor stimulation and resting-state functional hypoconnectivity in a widespread brain network including the hypothalamus [115]. Conversely, hypothalamic activity positively correlated with measures of typical social behavior in rats not responding to exposure to valproic acid in utero, another animal model of ASD [116]. 

In humans, hypoactivation of the hypothalamus and reduced oxytocin secretion has been observed in eating disorders [117,118]. However, to date, a clear demonstration of any relationship between hypothalamic activity and OT release at central and peripheral level in humans is still lacking. Furthermore, the limited spatial resolution of the considered studies does not permit to correctly attribute hypoactivation to single hypothalamic nuclei, all having diverse functions in the autonomic and central nervous system.

Reduced activation of the hypothalamus was also frequently associated with decreased amygdala activity in both ASD and carriers of OXTR rs53576A allele, in particular during face processing [64,86]. These findings are consistent with several previous studies reporting decreased amygdala activation during face perception in ASD [119,120]; nevertheless, opposite findings were also reported, but they were supposedly ascribable to longer gaze fixation time and higher anxiety level of individuals with ASD [58,121,122]. 

Hypothalamic nuclei can be controlled directly by the amygdala through the amygdalofugal pathway and the stria terminalis, and indirectly through the bed nucleus of the stria terminalis, which mediates stimulation of the hypothalamic-pituitary-adrenal axis. Projections of the central amygdala to the hypothalamus and brainstem can directly trigger autonomic fear responses [123]. Stimulation of amygdaloid OXT receptors is then assumed to inhibit these efferences’ activity so as to decrease aversive responses to socially-relevant stimuli [20,124,125], which would be increased in case of diminished amygdala activation. Accordingly, increased hypothalamic activity and amygdala deactivation appear to mediate initiation and consolidation of social relationship in healthy individuals. Such a reverse activation pattern of the hypothalamus and amygdala has been associated with several social behaviors such as other people’s trust and trustworthiness [126] and mother–infant and pair bonding [127,128,129,130], as well as visual processing of personally known faces including same-sex sibling and best friend with respect to unknown faces [131]. 

Prosocial behavior can be enhanced by hypothalamic through the modulation of two complementary responses: enhancement of social stimuli saliency processing and reduction of fear and avoidance behavior, both mechanisms being strictly dependent on amygdala activity [132,133]. Notably, AVP and OT have opposite modulatory effects on fear and anxiety-related behavior: the former by enhancing sympathetic responses such as stress level, anxiety, aggressiveness and boosting fear memory consolidation, the latter by acting on complementary parasympathetic responses that facilitate prosocial attitude and interactions as well as extinction of conditioned avoidance responses. These opposite regulatory neurophysiological processes result from activation of distinct elements of an inhibitory network within the medial part of the amygdala, and consecutive integration of different afferences to the central amygdala into a modulatory output to the hypothalamus and brainstem for appropriate anxiety and fear responses [134]. 

In addition, the hypothalamus can significantly influence socioemotional responses through a complex network that includes widely distributed, mostly bi-directional, neural connections to other brain regions. The hypothalamus is interconnected with basal forebrain areas such as the periamygdaloid region and the septal nuclei and other brainstem nuclei through the medial forebrain bundle, which mediates top-down modulation of both somatic and visceral activity by the forebrain and limbic system, as well as bottom-up influences of higher brain activity by internal organs and bodily interoceptive signals. 

Previous studies reporting hypothalamic activation concurrent to other socioemotional-related brain regions indeed indicated widespread interactions of the hypothalamus with emotional, motivational and social brain centers [132,135,136,137,138]. However, the still scarce evidence of functional connectivity of the hypothalamus in both NT and ASD individuals, which might also be partly dependent of the variable association between hypothalamic spiking activity and oxytocin release, do not permit to clarify how this region interacts during typical and atypical socioemotional behavior. 

Indirect indications about alterations of functional connectivity emerge from studies on healthy carriers of risk genetic mutations for ASD. Tost et al. (2010) measured increased structural and functional connectivity during face perception between hypothalamus and amygdala in OXTR risk allele carriers, suggesting a dysfunctional coupling underlying inappropriate responses to socially-relevant stimuli, although the actual nature of their interactions remains unknown. In addition, the same authors observed a negative coupling between hypothalamus and dorsal anterior cingulate and paracingulate cortex resulting from respectively decreased and increased activity [64]. Direct projections of the anterior cingulate cortex (ACC) to the hypothalamus have been demonstrated in both animals and humans [139,140]. Interestingly, concurrent increased activity in the paracingulate cortex and in the septal area, including the hypothalamus, has been associated with unconditional trust towards other people [126]. Maladaptive changes in trusting behavior, for instance after repeated violations of trust, consequent to exogenous administration of oxytocin, have been associated with increased ACC activity and decreased amygdala and midbrain activation [141]. The anticorrelation between the hypothalamus and ACC might then result from exaggerated ACC inhibitory activity of the hypothalamic nuclei preventing adaptive social behavior. The ACC is an important regulatory center that, through direct projections to the amygdala, insula, ventral striatum, hypothalamus and brainstem [140] can control socioemotional responses. Remarkably, it has been proposed that the medial prefrontal cortex, including ACC, would encode abstract representation of social experiences [142] permitting to predict and guide social goal-directed behavior based on social prediction error [143,144]. In line with this assumption, ACC connections with brain regions related to emotion and reward such as OFC, ventral and dorsal striatum, amygdala, insula and hypothalamus would then support generation of active inferences of affective, interoceptive and reward values [145,146] of socioemotional responses, as well as the minimization of prediction error between expected and actual behavioral outcome, the latter seemingly compromised in ASD [9,147].

### 3.3. Relevance of Hypothalamic Alterations in Healthy Carriers of Genetic Risk Variation in OT Receptors

Structural and functional alterations of the hypothalamus in individuals with polymorphisms of the OXTR gene are intriguing considering the increasing evidence indicating their relationship with ASD [79]. For instance, the OXTR rs53576A and recently the rs2268498 were associated with ASDs in both Asian and Caucasian populations [76,77,148,149]. Despite some inconsistency of the studies linking OXTR rs53576 variant with impaired socioemotional traits and behavior [150], the rs53576 and rs2254298 OXTR single nucleotide polymorphisms were shown to correlate with increased severity of social deficits in ASD, and less with social deficit in ADHD, thus indicating a differential relationship between this neuropeptide receptor gene allele and the social phenotype [80]. A metanalysis on the relationship between the OXTR rs53576 variant and human sociality indicated a clear influence of this OXTR polymorphism on individual psychological traits related to social responses to other people (for instance extraversion, empathy, and social loneliness) [151]. In short, neuroimaging findings in healthy carriers of OXTR rs53576A and OXTR rs2254298A genotypes indicate that alterations in the expression, and possibly function, of OXTR gene might be related to abnormal morphofunctional characteristics of the hypothalamus in ASD. However, it is conceivable that other OT signaling genes, such as the structural gene for OT (OT/neurophysin-I) [152] and gene for OT secretion (CD38) [153] that along with the OXTR have been frequently linked to human social behavior [154], might also contribute to structural and functional brain alterations in ASD. 

## 4. Conclusions

**Figure 1:**
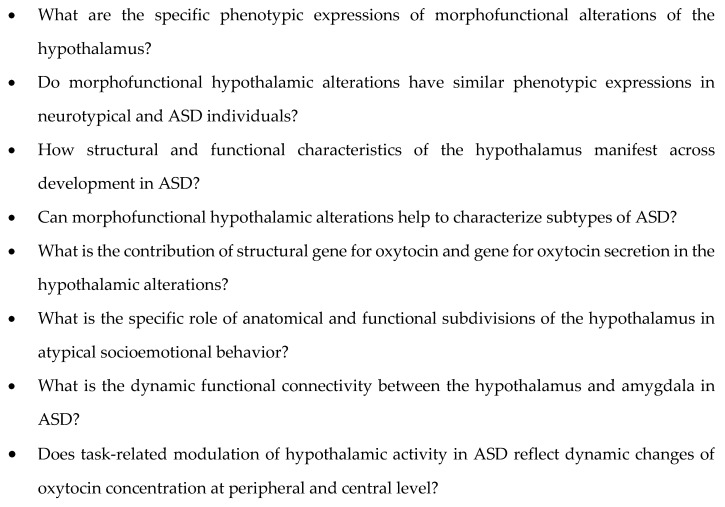
Questions for future research.

The few studies that have thus far observed, directly or indirectly, a relationship between the hypothalamus and ASD indicate both structural and functional alterations. However, considering the paucity of current investigations, further well-defined studies are strongly needed to clarify morphological and functional properties of hypothalamic nuclei and their complex functional exchanges with cortical and subcortical networks during socioemotional behavior in ASD (Figure 1).

Current neurophysiological investigations on the role of the hypothalamus in typical and atypical human social behavior have been likely hindered by several limitations related to the experimental methodology and MR signal acquisition techniques, resulting in a surprising disregard of its essential contribution. Designing protocols that permit to assess neural activity during realistic and entraining social scenarios, with rigorous control of experimental variables, for both NT and ASD individuals, is particularly challenging. In addition, MRI acquisition schemes adopted in most of previous anatomical and functional brain investigations in ASD were not specifically tailored for the hypothalamus. Neuroimaging of the small hypothalamic nuclei is certainly arduous as needs very high spatial resolution to clearly delineate their functional subdivisions and at the same time it requires prevention of potential partial volume effects, compensation for signal-dropouts occurring in ventromedial subcortical regions and correction for distortions generated by neighboring ventricles and blood vessels. Nevertheless, extraordinary progresses in high-field and ultra-high-field MRI techniques indicate feasibility of high-resolution structural [155,156] and functional [157,158] imaging of the human hypothalamus, and might then valuably support the elucidation of morphological and functional properties of this region in typical and atypical socioemotional behavior. Ultimately, greater understanding of the human hypothalamic morphology and functions is essential not only for the comprehension of socioemotional behavior but also in relation to the direct implication of hypothalamic neuropeptides in synaptic activity and plasticity [37], and neurogenesis [159], that may considerably impact the still obscure pathophysiology of ASD.
